# EVALUATION OF NUTRITIONAL STATUS IN PATIENTS WITH CYSTIC FIBROSIS ACCORDING TO AGE GROUP

**DOI:** 10.1590/1984-0462/;2019;37;1;00007

**Published:** 2018-08-09

**Authors:** Lenycia de Cassya Lopes Neri, Denise Pimentel Bergamaschi, Luiz Vicente Ribeiro Ferreira da Silva

**Affiliations:** a Universidade de São Paulo, São Paulo, SP, Brasil.

**Keywords:** Cystic fibrosis, Nutrition, Child, Adolescent, Fibrose cística, Nutrição, Criança, Adolescente

## Abstract

**Objective:**

To evaluate the nutritional profile of the population assisted at a reference center for cystic fibrosis treatment.

**Methods:**

Cross-sectional study including patients with cystic fibrosis assisted at a pediatric reference center in São Paulo, Brazil, in 2014. All patients attending regular visits who agreed to participate in the study were included. A questionnaire on dietary habits (24-hour diet recall) and socioeconomic characteristics was applied. Anthropometric data (compared with the reference from the World Health Organization, 2006 and 2007) and pulmonary function data were collected from medical records. Patients were stratified into age groups for statistical analysis.

**Results:**

A total of 101 patients were included in the study (59.4% male, 86.4% Caucasian), with median age of 10 years old. Most patients (n=77; 75%) were classified as eutrophic, but lower values of body mass index (BMI) Z scores were observed in schoolchildren and adolescents. The proportion of underweight patients was 10% (n=2) among preschoolers and 35% (n=6) of the school age group. Dietary intake was adequate, and the use of only two supplements (medium chain triglycerides and complete powdered supplement) was associated with adequacy of macronutrient intake. The socioeconomic characteristics did not show any statistical association with the nutritional state or nutritional consumption. Lung function was not significantly different between neither adolescents nor individuals with worse nutritional status in this sample.

**Conclusions:**

Most of the patients presented adequate nutritional status and adequate consumption of calories and macronutrients, indicating appropriate nutritional management. New studies focusing on preschool children should be performed to assess if it is possible to reduce the nutritional risk of patients with cystic fibrosis at older ages.

## RESUMO

**Objetivo:**

Avaliar o perfil nutricional da população atendida em centro de referência em fibrose cística.

**Métodos:**

Estudo transversal incluindo pacientes com fibrose cística de um centro pediátrico de referência de São Paulo, em 2014. Todos os sujeitos que concordaram em participar do estudo foram incluídos. Foi aplicado um questionário sobre hábitos alimentares (recordatório de 24 horas) e características socioeconômicas. Dados antropométricos (comparados com referencial da Organização Mundial da Saúde de 2006 e 2007) e função pulmonar foram coletados do prontuário. Os integrantes da pesquisa foram estratificados em faixas etárias para análise estatística.

**Resultados:**

Dos 101 pacientes incluídos no estudo, 59,4% eram masculinos, sendo a maioria caucasiana (86,4%), com mediana de idade de 10 anos. A maioria dos pacientes (n=77, 75%) foi classificada como eutrófica, mas valores menores de escore Z de índice de massa corpórea (IMC) foram observados em escolares e adolescentes. A proporção de pacientes com peso abaixo do esperado foi de 10% (n=2) na faixa etária pré-escolar e de 35% (n=6) em escolares. O consumo alimentar mostrou-se adequado, e somente dois suplementos (triglicérides de cadeia média e suplemento em pó completo) tiveram utilização ligada à adequação da ingesta de macronutrientes. Características socioeconômicas não apresentaram associação com o estado nutricional ou com o consumo nutricional. Valores de função pulmonar não apontaram diferença significante em adolescentes nem em indivíduos com pior estado nutricional no grupo estudado.

**Conclusões:**

A maioria dos pacientes apresentou estado nutricional e consumo alimentar adequados, indicando bom manejo das ações nutricionais. Novos estudos com foco em pré-escolares devem ser realizados para avaliar se é possível reduzir o risco nutricional de pacientes com fibrose cística em idades posteriores.

## INTRODUCTION

Cystic fibrosis is an autosomal recessive inherited disorder which leads to changes in the transportation of ions and water in cells of the respiratory gastrointestinal, hepatobiliary, reproductive systems and sweat glands.^1^ ^,^ ^2^

In the digestive system, for most patients, cystic fibrosis is manifested as exocrine pancreatic insufficiency, changes in intestinal motility and excessive presence of mucus in the enterocytes. All of these factors cause the poor digestion of fats, proteins and carbohydrates, and consequently, poor absorption. The clinical manifestation consists of chronic diarrhea, with voluminous, fatty, pale stool, with a characteristic smell, which, if not treated properly, leads to protein-energy malnutrition.^3^

The respiratory condition represents the main cause of mortality among patients, with chronic and recurring infections and progressive pulmonary lesions.^4^ ^,^ ^5^ ^,^ ^6^ ^,^ ^7^ Many studies have observed the close correlation between nutritional profile and respiratory conditions, and a better nutritional status can favor the patient’s prognosis.^8^ ^,^ ^9^

Therefore, the objective of this study was to assess the nutritional profile of the population assisted at a reference center for the treatment of cystic fibrosis.

## METHOD

This is a cross-sectional study including all patients with cystic fibrosis (diagnosed by two chloride sweat tests, with values indicating the disease)^10^ assisted at the outpatient clinic of Instituto da Criança (ICr), at Hospital das Clínicas in the Medical School of Universidade de São Paulo (FMUSP), from January to August, 2014. 

ICr is a tertiary, public, pediatric, university hospital located in the West zone of São Paulo, caring for children and adolescents aged between 0 and 19 years, especially by the Unified Health System (SUS). Care is provided by multidisciplinary teams, and the interval between appointments ranges according to the severity of the patient’s condition, but, in general, every two or three months. 

After the explanation about the research, the parents or tutors manifested accepting to participate in it by signing an informed consent form. The investigation was approved by the Ethics Committee of the institution (protocol from the Research Ethics Committee of Instituto de Medicina Tropical de São Paulo - CPE-IMT=000266). The only exclusion/loss criteria of the study were patients who did not accept being part of it, or those who missed scheduled appointments in the collection period.

A questionnaire was applied by the nutritionist to the parents (tutors) and patients (adolescents), including socioeconomic characteristics (income, number of people in the household) and dietary intake (24-hour diet recall, use of dietary supplements). In the analysis of the diet recall on the day prior to the appointment, only the cases with a routine dietary intake were included; those patients with an extraordinary day or acute pulmonary exacerbation were excluded (according to criteria from the medical appointment). The choice to collect a single diet recall was an attempt to minimize seasonal differences, by increasing data collection, and flaws in three-day dietary records. Family income (obtained in Reais) was transformed in categories of more or less than one minimum wage per capita. 

Anthropometric data (weight, height), pulmonary function and radiological clinical score were collected from the medical charts. When the charts did not have the described data, patients were excluded from the corresponding analysis. Anthropometry was carried out in the medical appointment in a standardized manner (weight in a n electronic scale, without clothes, or only underwear, height was measured by a stadiometer, or wooden ruler for infants). Pulmonary function data (spirometry) were obtained from the Brazilian Record of Cystic Fibrosis (patients aged ≥6 years, best result of the year), and expressed in Z score of the forced expiratory volume of the first second (FEV1), using the reference equation by Stanojevic et al.^11^ The general clinical status of the patient was measured by the Shwachman-Kulczycki score, annually, on the month of birthday of the patient.^12^

Patients were classified in age groups; infants were those aged less than 2 years; preschoolers, aged between 2 and 5 years (incomplete), students, aged between 5 and 10 years (incomplete), and adolescents, aged more than 10 years.

Patients were classified for nutritional status according to the programs Who Anthro and WHO AnthroPlus, of the World Health Organization (WHO, Geneve, Switzerland, 2006 and 2007),^13^ obtaining the Z score data of weight for age, height for age, weight for height and body mass index (BMI) for age. For the classification of eutrophy in infants, weight for height values were used, and, in other age groups. BMI values for age were used; those considered adequate were the ones that obtained indexes between -1 and +1 of the Z score. Z scores lower than -1 were classified as below expected, and higher than +1, above expected. The range between scores from -1 to -2, even though considered as “eutrophic” by the WHO^13^, and in the “low weight surveillance”, by the Brazilian Society of Pediatrics,^14^ was classified in this study as below expected, due to the close relationship between the worse nutritional status with the decreased pulmonary function in cystic fibrosis.^8^ ^,^ ^9^

The data about the intake of calories and macronutrients were calculated by the program NutWin (Universidade Federal de São Paulo - Unifesp, São Paulo, Brazil),^15^ software that has a data base of home measurements for estimating the referred data. The analysis of the adaptation of dietary intake was carried out based on values established for each patient, by the Institute of Medicine,^16^ using the nutritional recommendations of healthy children, with an increase of 20% in the necessary calories (as a minimum basic parameter in cystic fibrosis), and recommended distribution for cystic fibrosis: 40% of the calories of carbohydrates; 15% of the calories of proteins; and 35% of the calories of lipids.^1^ ^,^ ^17^ The analysis considered the percentages of adaptation of the real intake in relation to the recommendations (100% of adequacy indicates the consumption was equal to the recommendations). The dietary supplements used were examined in the analyses of adaptation of macronutrients according to their nutritional characteristics (for example, the supplements medium-chain triglycerides (MCT) was only considered for the adaptation of calories and lipids), and the indicated age group.

The obtained data were tabulated in Excel, version 2010 (Microsoft, Washington, the United States), and analyzed by descriptive statistics (median, 25 and 75 percentiles), with software Statistical Package for the Social Sciences (SPSS) version 19 (IBM, New York, the United States) and Stata version 11 (Statacorp, Texas, the United States). Nonparametric tests were used (Kruskal-Wallis) for variables that did not have normal distribution. The analyses of associations between the categorical variables were carried out using the χ^2^ and the Fisher tests. For the analyses of continuous variables, with normal distribution, t-test and ANOVA were used. Significance level of 0.05 was considered for rejecting the null hypothesis.

## RESULTS

Data of 101 patients were collected from an approximate total of 138 being followed-up. The losses were a result of missed appointments (n=4), not accepting to participate in the study (n=1), appointments that were not scheduled in the collection period (n=8), not being cared for in the reference center (n=8), or being hospitalized (n=6). Demographic and respiratory function data collected from charts proved that the losses were not statistically different between the analyzed patients (data not shown).

Of the included patients, 59.4% were male (n=60), and most were Caucasian (86.4%), and median age for inclusion was 10 years (p_25_= 4.1; p_75_= 3.7). Median age at diagnosis was 8 months.

Most patients included in the study were classified as eutrophic, but lower values were observed in the age groups of students and adolescents (Table 1).

**Table 1:** Sociodemographic characteristics, anthropometric and clinical data, by age group of patients with cystic fibrosis included in the study (n=94).

	Infant (n=9)	Preschooler (n=23)	Student (n=18)	Adolescent (n=51)	p-value
Age of diagnosis (years)	0.16 (0.11 / 0.61)	0.45 (0.22 / 1.19)	0.42 (0.28 / 2.17)	1.22 (0.53 / 4.69)	0.007***
Family income (in Reais per capita)	708 (262 / 1250)	500 (270 / 1000)	792 (500 / 1481)	431 (294 / 859)	0.455
Z score W/H	0.68 (-0.35 / 1.48)	-0.24 (-0.72 / 0.27)	-0.78 (-1.16 / 0.43)	-	0.142
Z score W/A	-0.18 (-1.08 / 0.80)	-0.47 (-1.01 / 0.06)	-0.88 (-1.19 / 0.26)	-1.26 (-1.26 / -1.25)	0.517
Z Score H/A	-0.99 (-2.24 / 0.11)	-0.50 (-1.19 / -0.02)	- 0.52 (-0.78 / 0.06)	- 0.85 (-1.75 / -0.35)	0.057
Z Score BMI	0.85 (-0.09 / 1.48)	-0.20 (-0.70 / 0.24)	-0.85 (-1.52 / 0.54)	-0.50 (-1.10 / 0.48)	0.027*
Shwachman Kulczycki Score	90 (85 / 95)	85 (75 / 95)	80 (75 / 85)	75 (55 / 80)	<0.001**
Days of hospitalization in the past year	15 (3 / 82)	7 (3.38 / 9.75)	13 (2.5 / 39.8)	14 (7 / 49.5)	0.30
Z Score FEV1	-	-	-0.24 (-2.82 / 0.64)	-2.44 (-3.94 / -0.86)	0.199

Z Score W/H: Z score of weight for height index; Z score W/A: Z score of weight for age index; Z Score H/A: Z score of height for age index; Z score BMI: Z score of body mass index for age; Z Score of FEV1: Z score of forced expiratory volume in the first second; *ANOVA: post-hoc Bonferroni test, being p=0.049 only in the comparison of the age groups infant and adolescent, and the other non-significant comparisons; **ANOVA: post-hoc Bonferroni test, being p=0.001 for preschoolers and adolescents, p=0.012 for infants and students, p<0.001 for infants and adolescents, p=0.035 for students and adolescents, and other non-significant comparisons; ***ANOVA: post-hoc Bonferroni test, being p=0.049 in the comparison of age groups infant and adolescents, and other non-significant comparisons.

Concerning dietary intake, the consumption reported was considered adequate for most patients, even with excessive carbohydrates as a source of calories for most of them (Table 2). Regarding the use of supplements, there were no differences between the types of supplements in the different age groups, even if some supplements are indicated only for specific ages. There was higher frequency of use of supplements in general among students and adolescents. In the evaluation of the association between the consumption of supplements and the adaptation in the intake of macronutrients (categorized analysis to verify if it reaches the recommendation or not, using the χ^2^ test), the use of MCT was associated with the intake of lipids (p=0.010), and the use of complete powdered supplement, to the adequate intake of proteins (p=0.003) and lipids (p=0.010).

**Table 2:** Adequacy reported in the intake of macronutrients (percentage of the recommended value for patients with cystic fibrosis), according to age gorup of patients in the study (n=94).

	Infant (n=9)	Preschooler (n=23)	Student (n=18)	Adolescent (n=51)	p-value*
Calories	109% (86 / 120)	117% (97 / 125)	100% (78 / 130)	111% (84 / 137)	0.629
Carbohydrates	132% (62 / 165)	130% (93 / 159)	119% (96 / 156)	146% (111 / 179)	0.224
Proteins	88% (77 / 156)	134% (102 / 171)	111% (91 / 154)	107% (95 / 138)	0.816
Lipids	93% (65 / 107)	109% (92 / 132)	111% (60 / 128)	87% (71 / 112)	0.588

Values reported in median (P_25_-P_75_); *ANOVA test.

**Figure 1:**
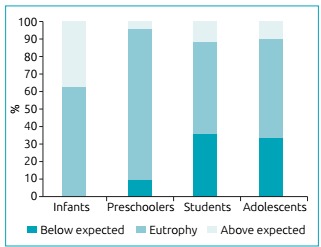
Classification of patients with cystic fibrosis according to nutritional status in Z score categories of body mass index (BMI) and age group: χ^2^ Pearson test and p=0.075.

By analyzing the categories of classification of the nutritional status, according to age groups, the observation was that whereas no infant presented weight deficit (Z W/H <-1), in the age group of preschoolers, about 10% of the patients presented a nutritional profile below expected (Z BMI <-1), and that proportion increased significantly among students (35.3%) and adolescents (33.3%); Figure 1). The nutritional status categories were not related to worse values of nutritional adequacy to the recommendations of calories and macronutrients, or the consumption of nutritional supplements.

**Figure 2:**
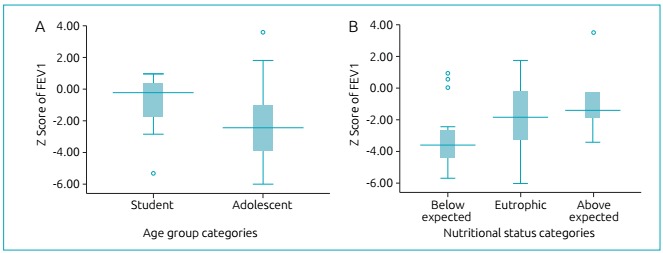
Z score values of expiratory forced volume in the first second (FEV1), according to the age groups of students and adolescents (A), according to nutritional status categories (B).

The pulmonary function data, available only for students and adolescents, show FEV1 values that are lower among adolescents (Figure 2), however, with no significant difference from the statistical point of view (p=0.199). By classifying the pulmonary function values according to the categories of nutritional diagnosis, reduced values are visualized in individuals with weight below expected, but, once again, without statistically significant differences (p=0.072).

Family income did not show any association with nutritional status, nor with the adequacy of energy and macronutrient consumption.

## DISCUSSION

This study showed that nutritional status of most patients of the analyzed center are eutrophic, with adequate dietary intake. The prevalence of below expected nutritional status, however, increased progressively with age, especially among the age groups of preschoolers and students. This indicates that the approaches addressed for the preschooler age group can be expressive to preserve the nutritional health of patients with cystic fibrosis.

The importance of nutrition in the health of patients with cystic fibrosis is well established in the literature,^17^ ^,^ ^18^ ^,^ ^19^ and several studies point adolescence as the most problematic age group in nutritional terms, with significant association to worse clinical and functional outcomes.^20^ This study, however, illustrates the relevance of na earlier action, focusing on preschoolers, phase that is knowingly difficult regarding diet.^21^

It is likely that families of preschoolers with cystic fibrosis face difficulties to support adequate nutritional support, and to maintain the diet for the treatment of pulmonary disease, aiming at guaranteeing ideal growth. At this stage, the infection and inflammation of the airways may occur in a little symptomatic way, resulting in structural damage.^22^ Some of the major challenges for the families are the inadequate dietary behaviors in this phase of childhood, which may lead to unwanted outcomes in goals of weight and height.^22^ On the other hand, sometimes the parent’s anxiety and poorly measured expectations lead to increasing diet refusal in the referred age group.^21^

Some examples of behavioral strategies are pointed out to increase the energy intake, and to improve growth at this age group: to present new foods 10-12 times, even if the child refuses them at first; to limit the time of meals from 15 to 30 minutes; to increase the calories of meals progressively; to take small meals in snack times; to compliment appropriate dietary behaviors; to prevent distractions in meals; to diversify the presentation of presentations; to avoid snacks; and to encourage the effective participation of the parents.^18^ ^,^ ^23^

Previous studies of nutrition in cystic fibrosis carried out in Brazil showed different results, possibly due to the variable availability of therapeutic and nutritional resources in the different Brazilian regions. In a cross-sectional study carried out in the Northeast, for instance, Pinto et al., in 2009, described worse nutritional status in patients with more unfavorable socioeconomic situation.^24^ In ICr, in a study carried out 12 years ago, the authors observed a higher proportion of patients with weight below expected through analysis of Z score in all of the anthropometric parameters and inadequate nutritional offers.^9^ This change in the nutritional profile of patients with cystic fibrosis is also observed in other countries^25^ and, is probably a result of factors such as earlier diagnosis, more strict control and more access to medicines and nutritional supplements.

Despite the diversity of options of available nutritional supplements for the study patients, the only nutritional supplements whose used was associated with the adequacy of consumption of macronutrients were MCT and complete powdered supplement, both being most frequently used in childhood.

This investigation has several limitations. The choice of a cross-sectional study design does not allow temporal or causal explanations in the complications involving nutritional status, so it is possible to estimate only higher prevalence rates of nutritional status deficit at more advanced ages. Besides, the analysis of dietary intake based only on a diet recall may mask flaws, even when excluding extraordinary days. The genotype and profile of colonization of the respiratory tract were not assessed, despite being known factors that affect the nutritional status. Another limiting factor is the sample number, because, by stratifying subjects by age group, some age groups had a reduced number of participants, which may justify some analyses without statistical significance (such as pulmonary function and nutritional status data).

The performance of anthropometric measurements that do not discriminate body composition may hide the diagnosis of lean mass deficit, with consequent pulmonary damage. Therefore, the presence of weight above expected in 12.9% of the sample may not be followed by the improvement in pulmonary function. A study from the reference center of cystic fibrosis in Greece showed similar prevalence to that found in our study, of patients with overweight and obesity,^26^ and another recent American study showed that the excess adiposity may reduce pulmonary function, even in patients considered eutrophic.^27^ It is possible that the recommendations for a hypercaloric fat-rich diet cause unwanted effects in some patients, so the recommendations and nutritional treatment should be individual.^28^

To sum up, the data presented in this study reveal that the work of a multidisciplinary team prior to the decline in nutritional status, and the availability of medicines and nutritional supplements are essential and bring results. On the other hand, it was possible to see the importance of a different approach for patients in the preschooler age group, who are going through a moment that is apparently sensitive and has an impact on nutritional outcomes. Additional educational strategies, including activities for the parents and children and graphic material, videos or digital media resources, can contribute with this scenario.^29^
